# In-home assessment of either topical fluralaner or topical selamectin for flea control in naturally infested cats in West Central Florida, USA

**DOI:** 10.1186/s13071-018-2995-1

**Published:** 2018-07-16

**Authors:** Michael W. Dryden, Michael S. Canfield, Cara Bocon, Letitia Phan, Emily Niedfeldt, Amanda Kinnon, Stanislaw A. Warcholek, Vicki Smith, Todd S. Bress, Nicole Smith, Kathleen Heaney, Christine Royal, Dorothy Normile, Robert Armstrong, Fangshi Sun

**Affiliations:** 1 Deptartment of Diagnostic Medicine/Pathobiology, Kansas State University, Manhattan, KS 66506 USA; 2 Animal Dermatology South, 7741 Congress St New Port Richey, Port Richey, FL 34653 USA; 3 Merck Animal Health, 2 Giralda Farms, Madison, NJ 07940 USA

**Keywords:** *Ctenocephalides felis felis*, Cats, Dogs, Fluralaner, Selamectin, Sarolaner, Flea control, Flea allergy dermatitis, Pruritus

## Abstract

**Background:**

An investigation was conducted in West Central Florida, USA to evaluate the efficacy of either topically applied fluralaner or topically applied selamectin to control flea infestations, minimize dermatologic lesions and reduce pruritus in naturally flea infested cats over a 12-week period. When dogs were present in the households, they were treated with either oral fluralaner (if household cats were treated with topical fluralaner) or oral sarolaner (if household cats were treated with topical selamectin).

**Methods:**

Thirty-one cats in 20 homes were treated once with fluralaner topical solution on day 0 and 18 dogs in these homes were administered a single fluralaner chewable. Twenty-nine cats in 18 homes were treated once monthly with a selamectin topical solution for 3 treatments and 13 dogs in these same homes were treated once monthly for 3 treatments with a sarolaner chewable. Fleas on cats were counted by flea combing, fleas on dogs were estimated using visual area counts and fleas in the indoor premises were assessed using intermittent-light flea traps. Blinded-assessments of feline dermatologic lesions were conducted monthly and pruritus severity was evaluated by pet owners.

**Results:**

A single topical application of fluralaner reduced flea populations on cats by 96.6% within 7 days and by 100% at 12 weeks post-treatment. This efficacy was significantly greater than selamectin treatment where single topical application reduced flea populations on cats by 79.4% within 7 days of initial treatment and 3 consecutive monthly treatments reduced flea populations by 91.3% at the end of 12 weeks. At the end of the 12-week study, all fluralaner-treated cats were flea-free and this was significantly greater than the 38.5% of selamectin treated cats that were flea-free. At the end of the study, fleas were completely eradicated (from cats, dogs and homes) in 95.0% of fluralaner treatment group homes, significantly greater than the 31.3% of selamectin/sarolaner treatment group homes with complete flea eradication. Owner reported cat pruritus was reduced similarly in both treatment groups. Significant improvements in dermatologic lesion scores were achieved by day 30 in fluralaner treated cats and by day 60 in selamectin treated cats.

**Conclusions:**

An in-home investigation in subtropical Florida found that 1 application of topical fluralaner eliminated flea infestations on cats and in homes significantly more effectively than 3 consecutive monthly doses of selamectin.

## Background

Flea allergy is considered the most common cause of dermatitis in dogs, and flea infestations in cats are also extremely common causes of irritation and pruritus leading to erythema, excoriations, papules and alopecia [1–4]. The flea species most commonly associated with these infestations in dogs and cats is the cat flea, *Ctenocephalides felis felis* [1, 2]. The cat flea is also a recognized intermediate host and vector of the common cestode *Dipylidium caninum*, as well as pathogens including *Rickettsia felis*, *Bartonella henselae* and *Mycoplasma haemofelis* [1–3]. Rapid and effective flea control is therefore, necessary to alleviate pruritus in cats and dogs and to help reduce the risk of disease agent transmission.

A new class of ectoparasiticides, the isoxazolines shows rapid residual speed of kill of fleas infesting dogs in both laboratory and field trials [5–9]. Recently a topical formulation of the isoxazoline fluralaner (Bravecto® Topical Solution, Merck Animal Health, Madison, NJ, USA) was introduced as an ectoparasiticide for dogs and cats [10–12]. This is the only topical isoxazoline formulation available to veterinarians in the USA at present. The extended retreatment interval of fluralaner, providing 12 weeks of protection against fleas, provides a new option to help veterinarians and cat owners deliver effective flea control. An extended duration of protection helps to improve owner compliance with veterinary parasite control recommendations [13].

The present study was conducted to evaluate the efficacy of topical fluralaner in naturally flea infested cats in subtropical Florida, USA. This area of Florida is known to carry a year round risk of intensive flea challenge. Selamectin (Revolution®, Zoetis, Whippany, NJ, USA), a flea control product with a monthly retreatment interval, was chosen as the positive reference control. The treatments were compared in their ability to eliminate existing flea infestations on cats, minimize pruritus and reduce dermatologic lesions and assess the impact on flea control within the home environment. Most homes in the area with cats also own dogs; therefore, co-habiting dogs were also treated for fleas. Dogs were treated with fluralaner in households with fluralaner treated cats. However, to match the monthly treatment recommendation of selamectin, dogs in these households were treated with the isoxazoline sarolaner (Simparica®, Zoetis, Whippany, NJ, USA). Sarolaner was selected for dogs based on the results of a prior flea efficacy trial conducted in the same area [7].

## Methods

### Inclusion criteria

Home owners with flea infested cats contacted the “Flea Team” through referrals from the Sunshine Animal Hospital, Tampa, FL, Animal Dermatology South, New Port Richey, FL, and advertisements on Facebook® and CRAIGSLIST®. Members of the team visited each residence, and 40 private residences were selected for inclusion in the study from May 17 through June 14, 2017. Selection criteria included: (i) ≥ five fleas in comb counts on at least one cat at the residence; (ii) ≥ five fleas trapped during a 16–24 h period in two intermittent-light flea traps; (iii) one to 10 healthy cats and dogs living at the private residence; (iv) cats spend ≥ 12 hours/day inside the residence; (v) homeowners agree not to use any other topical, oral or premise flea control products during the study; (vi) cats and dogs cannot be pregnant or nursing; (vii) all cats and dogs must be > 6 months of age and cats need to be at least 1.18 kg and dogs > 2 kg; and (viii) owners sign a consent form and fill out a questionnaire concerning pet habits, flea control history and personal observations concerning potential flea hosts around their residence.

### Flea population assessment

Flea infestation in indoor residence areas was assessed using two intermittent-light traps (MyFleaTrap™, Zantey Inc, Tallahassee, FL, USA) [14, 15]. Traps were placed one each in two rooms during 16- to 24-hour collection periods. Rooms were chosen based on where the cat(s) spent most of their time, or where fleas had been observed by owners. At each collection period traps were returned to the same location in the room. Species, number and sex of fleas collected on the adhesive-sheets of the traps were recorded.

Prior to initiating the study all personnel were trained in proper cat handling techniques using American Association of Feline Practitioners and American Animal Hospital Association recommended methods for proper and safe handling of cats. Fleas on cats were counted using a modified combing procedure due to time and safety constraints. Six regions were examined using 10 strokes of a standard flea comb per region: (i) back of the head behind the ears and extending along the dorsal midline to the tail; (ii) left and (iii) right side from axillary region to posterior aspect of the cat’s body, including hair on posterior of each leg; (iv) ventral aspect from chest to inguinal region; (v) ventral neck region from chin to chest; and (vi) top of the head between the ears. As fleas were collected they were placed in a plastic bag with collected hair. Fleas were counted and immediately placed back on the cat.

Fleas on dogs were estimated using a previously described area count methodology [16]. Fleas were counted in five areas on each animal; dorsal midline, tail head, left lateral, right lateral, and inguinal region. Due to the effects of large flea numbers on the accuracy of area counts, flea numbers in each of the five areas was capped at 50; therefore, the maximum total area flea count was 250.

All on-animal and premises flea counts were conducted ± 1 day on days 0, 7, 14, 21, then once between days 28–30, 40–45, 56–60 and 82–86. Personnel conducting pet and premises flea counts were not blinded to treatment groups.

### Evaluation of pruritus and dermatologic lesions

Pet owners assessed the severity of pruritus of dogs(s) in each home during each scheduled visit using a previously validated and described non-numeric scale [17, 18]. In each home one owner completed the assessment. There is no equivalent validated technique for pruritus assessment in the cat; therefore, the owner-assessed pruritus visual analogue score (PVAS) used in this study was a modification of a previously published feline pruritus visual analogue score [19]. The severity of pruritus of the qualifying cat(s) in each home was similarly evaluated at each visit by the owner. Owners rated the pruritus level of the qualifying cat(s) using a non-numeric scale on a data capture form with descriptions of increasing severity. The owner pruritus rating was then numerically assessed as previously described for the dog [17, 18].

In both assessments owners did not see, nor were they informed of the numerical score given to their dog(s) or cat(s). In households with more than one owner, the same owner was required to assess the pruritus level of the pet(s) throughout the study.

Blinded clinical dermatologic observations were made on days 0, 30, 60, and 84 of the study (± 3 days) of all qualifying cats resident in the homes. The extent and severity of dermatologic lesions were assessed using the validated scale, Scoring Feline Allergic Dermatitis (SCORFAD) [19]. For this assessment 10 body zones were assessed for excoriations, miliary dermatitis, eosinophilic plaques, and self-induced alopecia using a score from 0–4 for each category and lesion type [19]. The percentage reduction in SCORFAD from baseline was previously determined to be the most valid assessment of clinical response and has been proposed as an assessment tool in feline hypersensitivity dermatitis [18].

### Treatment groups

Qualifying homes and all pets in that household were randomly allotted to 1 of 2 treatment groups on day 0. Household entry numbers (1–40) were assigned a random number by Excel (Excel 2016, Microsoft, Redmond, WA) and blocked into groups of 2. The highest random number within each block was assigned to group 1 and the lowest to group 2.

In treatment Group 1 cats were administered a topical fluralaner solution (Bravecto® Topical Solution; Merck Animal Health, Madison, NJ, USA) at the recommended labeled dose (minimum 40 mg/kg). While this study was focused on cats, dogs resident in these households were administered an oral fluralaner chew (Bravecto® Chewable Tablets; Merck Animal Health, Madison, NJ, USA) at the recommended labeled dose (minimum 25 mg/kg).

In treatment Group 2 cats were administered a topical selamectin solution (Revolution®; Zoetis, Whippany, NJ, USA) at the recommended labeled dose (minimum 6 mg/kg). While this study was focused on cats, any dogs resident in these households were also administered an oral sarolaner chew (Simparica®; Zoetis, Whippany, NJ, USA) at the recommended labeled dose (minimum 2 mg/kg).

All animals were weighed on a calibrated scale prior to treatments and products were administered according to product labeling by study personnel. Fluralaner was administered once on day 0. Selamectin and sarolaner were administered three times; once on study day 0; once between days 28–30 and finally between days 56–60. All dogs and cats living at a residence were administered group appropriate treatment and no alternative flea treatments were used during the study on pets or premises. During this study no corticosteroids, antihistamines, antibiotics or medicated shampoos were used to alleviate pruritus or skin lesions. No restrictions were placed on the animals regarding swimming, non-insecticidal baths or movement outdoors.

This study was conducted without a placebo control group because the heavy and constant flea challenge experienced by cats and dogs in subtropical Florida would make inclusion of a non-treated group inhumane. Withholding flea adulticide treatment would be detrimental to the health and welfare of the pets and potentially also to humans in these households.

### Data analysis

The animal and trap flea count data were transformed prior to analysis using the Y= log_e_(x+1) transformation. The log-transformed flea counts on animals were analyzed by a mixed linear model with repeated measures including treatment, day, treatment*day as the fixed effects; and household, and animal as random effects. The log-transformed flea counts in traps were analyzed by a mixed linear model with repeated measures including treatment, day, and treatment*day as the fixed effects and household as random effect.

A Kenward-Rogers adjustment was used to determine the denominator degree of freedom for hypothesis. Akaike’s information criterion (AIC) was used as the criterion to select the covariance structure for repeated measures. The dermatology, pruritus and SCORFAD scores were analyzed by the same mixed linear model with repeated measures as that for the flea counts on dogs. Percentages of animals without fleas were analyzed and compared using Fisher’s exact test. All comparisons were made between treatment groups on each data collection day and also between each collection day and the baseline (day 0) values within each treatment group.

A two-tailed t-test was used for the comparison and significance was declared when *P* < 0.05; 90% confidence intervals were constructed for the differences between treatment groups for the equivalence declaration. The primary software was SAS version 9.3 (SAS® Language: Reference, Version 9.3, SAS Institute Inc., Cary, NC, USA).

Percent control of flea counts were calculated using geometric means with Abbott’s formula:

$$ \mathrm{Efficacy}\kern0.5em \left(\%\right)\kern0.5em =\kern0.5em 100\kern0.5em \times \kern0.5em \left({\mathrm{M}}_{\mathrm{B}}\kern0.5em -\kern0.5em {\mathrm{M}}_{\mathrm{C}}\right)\kern0.5em /\kern0.5em {\mathrm{M}}_{\mathrm{B}} $$

where M_C_ is the geometric mean number of fleas on flea count day and M_B_ is the geometric mean number of live fleas count on baseline.

Percent reduction of clinical scores were calculated using arithmetic means with Abbott’s formula:

$$ \mathrm{Efficacy}\kern0.5em \left(\%\right)\kern0.5em =\kern0.5em 100\kern0.5em \times \kern0.5em \left({\mathrm{M}}_{\mathrm{B}}\kern0.5em -\kern0.75em {\mathrm{M}}_{\mathrm{C}}\right)\kern0.5em /\kern0.5em {\mathrm{M}}_{\mathrm{B}} $$

where M_C_ is the arithmetic mean of clinical scores on score collection day and M_B_ is the arithmetic mean of clinical scores on baseline.

## Results

Initially 40 private residences were enrolled in the study, although four residences in the selamectin-sarolaner treatment group did not complete the study. Two homes were dropped within the first 2 weeks because of severe cockroach infestations and data from these households were excluded. The owners in one residence moved because of the infestation severity and owners in the other residence had a professional pest management company spray their entire home with an insecticide/insect growth regulator combination. The third residence, with two enrolled cats, was lost after the 28–30-day assessment because the home was vacated after a small electrical fire. The fourth home, with one enrolled cat and one dog, was lost following the day 56–60-day appointment because the owners moved to a new home. Data from these two residences and their enrolled pets were included in analysis up to the point they were lost from the study.

Additionally, there were three times during the study when data was not collected at a single counting period on an individual pet or an entire home because the animal was unavailable, or the owners were not at home that week. On day 7 data were collected from 19 of 20 homes in the fluralaner group and on day 14 data were collected from 17 of 18 homes in the selamectin/sarolaner group.

In the 20 homes in the fluralaner treatment group, 31 cats (mean 4.7 kg; range 2.9–6.7 kg) were officially enrolled on day 0. These cats were administered a mean topical dose of 58.9 mg/kg (range 40.7–86.3 mg/kg) fluralaner. There were also 18 dogs (mean 24.7 kg; range 4.8–37.7 kg) officially enrolled on day 0 and they were administered a mean oral dose of 36.6 mg/kg (range 26.5–52.4 mg/kg) fluralaner. Additionally, there were 28 cats and 14 dogs that were treated but did not meet qualifying criteria. These animals had < 5 fleas; spent the majority of their time outside the residence; or could not be safely handled. These households had a total of 91 pets (59 cats and 32 dogs) treated with fluralaner.

In the 18 homes that remained in the selamectin/sarolaner treatment group for at least 4 weeks, 29 cats (mean 4.8 kg; range 2.4–9.2 kg) and 13 dogs (mean 22.2 kg; range 2.5–47.2kg) were enrolled. On day 0, cats were topically treated with a mean dose of 10.4 mg/kg (6.6–18.4 mg/kg) selamectin and dogs were orally administered a mean dose of 2.8 mg/kg (2.0–3.8 mg/kg) sarolaner. There were an additional 12 cats and 12 dogs in these residences that did not qualify for inclusion in the study for reasons previously described. These households had a total of 66 pets (41 cats and 25 dogs) administered group appropriate treatments.

Pre-treatment geometric mean flea counts for cats in both groups (Table 1) and dogs in both groups (Table 2) were completed on day 0. There were significantly more pre-treatment fleas on dogs in the sarolaner treatment group than in the fluralaner treatment group on day 0 (Table 2). Flea control efficacy of fluralaner on treated cats, calculated based on flea counts, was significantly superior to selamectin (Table 1). Within one week following the application of fluralaner topical solution to cats, flea counts were significantly reduced by 96.6% (Table 1), whereas flea counts on cats administered selamectin were significantly reduced by 79.4% (Table 1) in the first week after treatment. By days 28–30, mean flea counts on fluralaner treated cats were reduced by 98.5% and reductions remained between 99.2–100% for the remainder of the 12-week study following a single topical dose (Table 1). Following 3 monthly applications of selamectin, flea populations were reduced by 91.31% by days 82–86. (Table 1). Post-treatment flea counts were significantly different from day 0 counts at every time point for both fluralaner and selamectin treated cats (Table 1). Mean flea counts on fluralaner treated cats were significantly lower than mean flea counts on selamectin treated cats at every post-treatment assessment (Table 1).

**Table 1:** Flea counts on naturally infested cats in households in Florida before and after treatment with either a single topical dose of fluralaner or three consecutive monthly doses of selamectin

Treatment group	No. of cats on day 0		Days post-treatment							
			0	7	14	21	28–30	40-45	56-60	82-86
Fluralaner^1^	31	Geomean flea count^3^	11.4^a,x^	0.4^b,y^	0.3^b,y^	0.1^b,y^	0.2^b,y^	0.1^b,y^	0.1^b,y^	0.0^b,y^
		Range	(5–63)	(0–4)	(0–4)	(0–3)	(0–2)	(0–2)	(0–3)	(0–0)
		% control^4^		96.6	97.3	99.3	98.5	99.5	99.2	100
		% (#) cats with no fleas	0.0^a,x^ (0/31)	69.0^b,y^ (20/29)	71.0^b,y^ (22/31)	93.5^b,y^ (29/31)	80.6^b,y^ (25/31)	93.5^b,y^ (29/31)	90.3^b,y^ (28/31)	100^b,y^ (31/31)
Selamectin^2^	29	Geomean flea count^3^	12.7^a,x^	2.6^a,y^	3.2^a,y^	3.6^a,y^	2.8^a,y^	0.8^a,y^	1.1^a,y^	1.1^a,y^
		Range	(5–44)	(0–23)	(0–29)	(0–50)	(0–35)	(0–14)	(0–11)	(0–8)
		% control^4^		79.4	74.6	71.3	77.8	93.8	91.0	91.3
		% (#) cats with no fleas	0.0^a,x^ (0/29)	24.1^a,y^ (7/29)	28.6^a,y^ (8/28)	17.2^a,x^ (5/29)	17.2^a,x^ (5/29)	37.0^a,y^ (10/27)	44.4^a,y^ (12/27)	38.5^a,y^ (10/26)

^1^In the fluralaner group cats were treated once topically on day 0 (Bravecto® Topical solution; Merck Animal Health, Madison, NJ, USA)

^2^In the selamectin group cats were treated on day 0 and once between days 28–30 and 56–60 (Revolution®; Zoetis, Whippany, NJ, USA)

^3^Geometric mean numbers of fleas in comb counts

^4^{(Day 0 geometric mean animal area flea counts – day x geometric mean animal area flea counts) / day 0 geometric mean animal area flea counts)} × 100

^a,b^Geometric mean flea counts in a column with unlike letter superscripts are significantly different (*P* < 0.001; │*t*│≥ 3.70)

^x,y^Geometric mean flea counts in a row with unlike letter superscripts are significantly different from day 0 (*P* < 0.001; │*t*│≥ 7.14)

^a,b^Percent of flea-free cats in a column with unlike letter superscripts are significantly different (*P* < 0.002; Fisher’s exact test)

^x,y^Percent of flea- free cats in a row with unlike letter superscripts are significantly different from day 0 (*P* < 0.01; Fisher’s exact test)

**Table 2:** Flea counts on naturally infested dogs in households in Florida before and after treatment with a single oral dose of fluralaner or three consecutive monthly doses of sarolaner

Treatment group	No. of dogs on day 0		Days post-treatment							
			0	7	14	21	28–30	40–45	56–60	82–86
Fluralaner^1^	18	Geomean flea count^3^	26.9^b,x^	0.1^a,y^	0.3^a,y^	0.1^a,y^	0.0^a,y^	0.1^a,y^	0.0^a,y^	0.0^a,y^
		Range	(5–131)	(0–1)	(0–3)	(0–2)	(0–0)	(0–2)	(0–0)	(0–0)
		% control^4^		99.7	99.0	99.8	100	99.6	100	100
		% (#) dogs with no fleas	0.0^a,x^ (0/18)	88.9^a,y^ (16/18)	72.2^a,y^ (13/18)	94.4^a,y^ (17/18)	100^a,y^ (18/18)	88.9^a,y^ (16/18)	100^a,y^ (18/18)	100^a,y^ (18/18)
Sarolaner^2^	13	Geomean flea count^3^	37.9^a,x^	0.1^a,y^	0.1^a,y^	0.2^a,y^	0.1^a,y^	0.0^a,y^	0.0^a,y^	0.0^a,y^
		Range	(10–171)	(0–1)	(0–1)	(0–2)	(0–1)	(0–0)	(0–0)	(0–0)
		% control^4^		99.7	99.8	99.4	99.9	100	100	100
		% (#) dogs with no fleas	0.0^a,x^ (0/13)	84.6^a,y^ (11/13)	91.7^a,y^ (11/12)	76.9^a,y^ (10/13)	92.3^a,y^ (12/13)	100^a,y^ (13/13)	100^a,y^ (13/13)	100^a,y^ (12/12)

^1^In the fluralaner group dogs were treated once orally on day 0 (Bravecto chew; Merck Animal Health, Madison, NJ, USA)

^2^In the sarolaner group dogs were treated on day 0 and once between days 28–30 and 56–60 (Simparica chew; Zoetis, Whippany, NJ, USA)

^3^Geometric mean numbers of fleas in area counts

^4^{(Day 0 geometric mean animal area flea counts – day x geometric mean animal area flea counts) / day 0 geometric mean animal area flea counts)} × 100

^a,b^Geometric mean flea counts in a column with unlike letter superscripts are significantly different (day 0, *P* = 0.034; │*t*│ = 2.14 )

^x,y^Geometric mean flea counts in a row with unlike letter superscripts are significantly different from day 0 (*P* < 0.001; │*t*│ ≥ 22.83)

^a,b^Percent of flea- free dogs in a column with like letter superscripts are not significantly different (*P* > 0.284; Fisher’s exact test)

^x,y^Percent of flea- free dogs in a row with unlike letter superscripts are significantly different from day 0 (*P* < 0.001; Fisher’s exact test)

Following a single topical fluralaner dose, 80.6% (25/31) of treated cats had no fleas recovered in comb counts on days 28–30 and 100% (31/31) of cats were flea free at 12 weeks (Table 1) and the number of flea free cats was significantly different from day 0 pre-treatment counts at every post-treatment assessment (Table 1). The number of flea-free fluralaner treated cats was significantly greater than the number of flea-free selamectin treated cats at every post-treatment assessment (Table 1). The percentage of selamectin treated flea free cats was 17.2% (5/29) at 4 weeks and 38.5% (10/26) at 12 weeks (Table 1). The number of cats with no fleas collected in comb counts (flea-free cats) following selamectin administration was significantly different from day 0 pre-treatment counts on days 7, 14, 40–45, 56–60 and 82–86, but was not significantly different from pre-treatment counts on days 21 and 28–30 (Table 1).

Dogs treated orally with either one dose of fluralaner or 3 monthly doses of sarolaner had remarkably similar flea count reductions (Table 2). Within 7 days of treatment, area flea counts on dogs were reduced by 99.7% in both treatment groups and flea populations on dogs were reduced by 99.6–100% at every counting period from 4 weeks to the end of the 12-week study (Table 2). Flea counts on treated dogs were significantly reduced from day 0 counts at every post-treatment assessment in both treatment groups, and there were no post-treatment differences in flea counts or number of flea-free dogs between the two treatment groups (Table 2).

During the entire 12-week study, 3525 fleas were collected in intermittent light traps in 38 residences and all were identified as *C*. *f*. *felis*, the cat flea. On day 0, pre-treatment geometric mean flea collections were 32.4 (range 5–183) in the fluralaner group traps and 28.0 (range 5–152) in the selamectin/sarolaner treatment group traps (Table 3). Overall flea populations in premises were significantly reduced in both the fluralaner and selamectin/sarolaner treatment groups over the 12-week study, with flea trap counts reduced by 99.9% in the fluralaner group and 98.5% in the selamectin/sarolaner treatment group (Table 3). Reductions were similar for both groups at all post-treatment assessments except on day 21 when the mean flea count in fluralaner homes was significantly lower (Table 3).

**Table 3:** Fleas caught in intermittent-light flea traps in naturally infested homes in Florida before and after treatment of all dogs and cats in the homes with either a single administration of fluralaner or three consecutive monthly administrations of sarolaner (dogs) and selamectin (cats)

Treatment group		Days post-treatment								
			0	7	14	21	28–30	40–45	56–60	82–86
Fluralaner^1^	20 homes on study									
		Geomean flea count^3^	32.4^a,x^	6.3^a,y^	7.2^a,y^	1.6^b,y^	0.5^a,y^	0.2^a,y^	0.1^a,y^	0.04^a,y^
		Range	(5–183)	(0–113)	(0–156)	(0–19)	(0–6)	(0–5)	(0–1)	(0–1)
		% control^4^		80.4	77.9	95.0	98.4	99.4	99.8	99.9
	% (#) homes with 0 fleas in traps		0.0^a,x^ (0/20)	10.5^a,x^ (2/19)	20.0^a,x^ (4/20)	30.0^a,y^ (6/20)	70.0^a,y^ (14/20)	85.0^a,y^ (17/20	90.0^a,y^ (18/20)	95.0^a,y^ (19/20)
	% (#) homes with no fleas (traps, cats or dogs)		0.0^a,x^ (0/20)	15.0^a,x^ (3/20)	15.0^a,x^ (3/20)	30.0^b,y^ (6/20)	60.0^b,y^ (12/20)	75.0^b,y^ (15/20)	80.0^b,y^ (16/20)	95.0^b,y^ (19/20)
Selamectin/Sarolaner^2^	18 homes on study	Geomean flea count^3^	28.0^a,x^	4.7^a,y^	4.8^a,y^	4.2^a,y^	1.4^a,y^	0.04^a,y^	0.4^a,y^	0.4^a,y^
		Range	(5–152)	(0–195)	(0–93)	(0–22)	(0–1)	(0–14)	(0–39)	(0–12)
		% control^4^		83.1	82.7	85.0	95.1	99.9	98.8	98.5
	% (#) homes with 0 fleas in traps		0.0^a,x^ (0/18)	16.7^a,x^ (3/18)	17.6^a,x^ (3/17)	22.2^a,x^ (4/18)	44.4^a,y^ (8/18)	94.1^a,y^ (16/17)	82.4^a,y^ (14/17)	68.8^a,y^ (11/16)
	% (#) homes with no fleas (traps, cats or dogs)		0.0^a,x^ (0/18)	5.6^a,x^ (1/18)	16.7^a,x^ (3/18)	0.0^a,x^ (0/18)	5.6^a,x^ (1/18)	23.5^a,y^ (4/17)	29.4^a,y^ (5/17)	31.3^a,y^ (5/16)

^1^In the fluralaner group dogs were treated once orally on day 0

^2^In the selamectin/sarolaner group cats and dogs were treated on day 0 and again between days 28–30 and between days 56–60

^3^Geometric mean numbers of fleas recovered in two intermittent light flea traps averaged within households

^4^{(Day 0 geometric mean animal area flea counts – day x geometric mean animal area flea counts) / day 0 geometric mean animal area flea counts)} × 100

^a,b^Geometric mean trap flea counts in a column with unlike letter superscripts are significantly different (Day 21; *P*=0.032; │*t*│ = 2.18)

^x,y^Geometric mean trap flea counts in a row with unlike letter superscripts are significantly different from day 0 (*P* < 0.001; │*t*│ ≥ 5.57)

^a,b^Percent of flea- free traps in a column with like letter superscripts are not significantly different (*P* > 0.069; Fisher’s exact test).

^x,y^Percent of flea- free traps in a row with unlike letter superscripts are significantly different from day 0 (*P* < 0.020; Fisher’s exact test)

^a,b^Percent of flea- free homes in a column with unlike letter superscripts are significantly different (*P* < 0.021; Fisher’s exact test)

^x,y^Percent of flea- free homes in a row with unlike letter superscripts are significantly different from day 0 (*P* < 0.046; Fisher’s exact test)

Overall flea trap counts were similarly reduced in both treatment groups and there were similar numbers of homes where traps were flea free throughout the study (Table 3). The number of homes with flea free traps on days 7 and 14 was not different from day 0 in the fluralaner group and was not different from day 0 on days 7, 14 and 21 in the selamectin/sarolaner treatment group, likely due to continued emergence of flea stages developing in the household before the start of the study. At the 82–86 day count, 95.0% (19/20) of homes in the fluralaner treatment group and 68.8% (11/16) of homes in the selamectin/sarolaner treatment group had flea free traps (Table 3).

The ratio of female to male fleas collected in flea traps in homes with fluralaner-treated cats and dogs shifted over time. On day 0, prior to treatment, 58.0% of fleas collected in intermittent-light flea traps in fluralaner treatment group homes were female. At weeks 1, 2, 3, 4 and 6, females represented 62.3, 47.9, 38.7, 35.0 and 37.5%, respectively, of collected fleas. Both fleas collected on traps in fluralaner homes at week 8 and the single flea collected in a trap at week 12 were male. A different trend was observed in the selamectin/sarolaner treatment group homes. On day 0, prior to treatment, 51.2% of fleas collected in traps in these homes were female. At weeks 1, 2, 3, 4 and 6, females represented 54.3, 53.6, 48.7, 5.3 and 0.0%, respectively, of the fleas collected; however, the percentage of female fleas increased to 36.6% at week 8 and to 41.2% at week 12.

Total flea eradication was defined as a home with no fleas on cats or dogs, or in traps, and there were significantly more homes with total flea eradication in the fluralaner treatment group than in the selamectin/sarolaner group at all post-treatment assessments after the second week (Table 3). The proportion of homes with total flea eradication at 4 weeks post-treatment was 60.0% (12/20) in the fluralaner treatment group homes, significantly greater than the 5.6% (1/18) of selamectin/sarolaner treatment group homes. At the end of the 12-week study total flea eradication occurred in 95.0% (19/20) of fluralaner homes, significantly greater than the 31.3% (5/16) of selamectin/sarolaner homes (Table 3).

Cats in fluralaner and selamectin treatment groups had mean pruritus (PVAS) scores of 5.87 (range 1.1–10) and 6.62 (range 0.3–10), respectively on day 0 (Table 4). Owner assessed pruritus severity reduced significantly on all post-treatment days for both treatment groups (Table 4). Pruritus severity in fluralaner treated cats was significantly lower than selamectin treated cats on days 21 and 28–30 (Table 4). Both groups had similar improvements in pruritus severity scores from day 40 until the end of the study.

**Table 4:** Owner assessment of pruritus using a visual analogue scale (PVAS) for cats in homes naturally infested with fleas before and after treatment with either a single topical dose of fluralaner or three consecutive topical treatments with selamectin

Treatment group		Days post-treatment							
		0	7	14	21	28–30	40–45	56–60	82–86
Fluralaner^1^	# Cats	31	30	30	30	31	31	31	31
	Mean PVAS Score^3^	5.9^a,x^	2.6^a,y^	1.9^a,y^	1.1^b,y^	1.2^b,y^	1.0^a,y^	1.1^a,y^	0.9^a,y^
	SD	2.31	1.93	1.61	1.40	1.70	1.32	1.71	0.96
	Range	(1.1–10)	(0.0–8.2)	(0.0–5.2)	(0.0–5.0)	(0.0–6.3)	(0.0–5.1)	(0–6.7)	(0.0–2.7)
	% Reduction^4^		56.0	68.0	80.7	79.7	82.7	81.7	84.3
Selamectin^2^	# Cats	29	28	28	29	28	26	27	26
	Mean PVAS Score^3^	6.6^a,x^	3.1^a,y^	2.7^a,y^	2.9^a,y^	2.6^a,y^	1.3^a,y^	1.6^a,y^	1.6^a,y^
	SD	2.58	1.09	2.12	2.40	2.31	1.46	1.92	2.12
	Range	(0.3–10)	(1.0–5.1)	(0.0–8.0)	(0.2–8.8)	(0.0–9.3)	(0.0–4.7)	(0.0–7.5)	(0.0–6.7)
	% Reduction^4^		53.6	58.9	56.6	60.6	80.9	76.2	76.5

^1^In the fluralaner group cats were treated once topically on day 0 (Bravecto® Topical solution; Merck Animal Health, Madison, NJ, USA)

^2^In the selamectin group cats were treated on day 0 and once between days 28–30 and 56–60 (Revolution®; Zoetis, Whippany, NJ, USA)

^3^Arithmetic mean pruritus scores as assessed by cat owners using a PVAS scoring sheet

^4^{(Day 0 arithmetic mean PVAS score – day x arithmetic mean PVAS score) / day 0 arithmetic mean PVAS score)} × 100

^a,b^Arithmetic mean PVAS scores in a column with unlike letter superscripts are significantly different (*P* < 0.005; │*t*│ ≥ 2.882)

^x,y^Arithmetic mean PVAS scores in a row with unlike letter superscripts are significantly different from day 0 (*P* < 0.001; │*t*│≥ 8.680)

On day 0 of the study, blinded pre-treatment feline dermatology lesion scores were similar in both treatment groups (Table 5). Differences between groups post-treatment were not significant; however, the fluralaner-treated cats had significant improvements from baseline by day 30 and selamectin-treated cats were significantly improved from baseline by day 60 (Table 5).

**Table 5:** Assessment of dermatologic lesions using a feline allergic dermatitis (SCORFAD) severity scale for cats naturally infested with fleas in homes in Florida and treated with either a single topical dose of fluralaner or three consecutive monthly doses of selamectin

Treatment group		Days post-treatment			
		0	30	60	84
Fluralaner^1^	No. of cats	31	31	31	31
	Mean SCORFAD Score^3^	7.5^a,x^	2.7^a,y^	1.3^a,y^	1.1^a,y^
	SD	8.39	3.96	1.77	1.47
	Range	(0–28)	(0–18)	(0–7)	(0–6)
	% Reduction^4^		63.6	82.8	85.3
Selamectin^2^	No. of cats	29	29	27	26
	Mean SCORFAD Score^3^	7.7^a,x^	5.6^a,x^	3.2^a,y^	3.4^a,y^
	SD	10.34	8.85	4.73	3.90
	Range	(0–50)	(0–35)	(0–21)	(0–13)
	% Reduction^4^		26.9	58.1	56.5

^1^In the fluralaner group cats were treated once topically on day 0 (Bravecto® Topical solution; Merck Animal Health, Madison, NJ, USA)

^2^In the selamectin group cats were treated on day 0 and once between days 28–30 and 56–60 (Revolution®; Zoetis, Whippany, NJ, USA)

^3^Arithmetic mean SCORFAD lesion score

^4^{(Day 0 arithmetic mean SCORFAD score – day x arithmetic mean SCORFAD score) / day 0 arithmetic mean SCORFAD score)} × 100

^a,b^Arithmetic mean SCORFAD lesion scores in a column with like letter superscripts are not significantly different (*P* > 0.058; │*t*│ ≥ 0.21).

^x,y^Arithmetic mean SCORFAD lesion scores in a row with unlike letter superscripts are significantly different from day 0 (*P* < 0.001; │*t*│≥ 3.35; day 30 Selamectin *vs* baseline *P* = 0.13; *t* = 1.53)

Client interviews showed that reservoir hosts for *C. f. felis* were commonly observed by pet owners on their properties. Many pet owners reported opossums (42.1%; 16/38), raccoons (57.9%; 22/38) and/or feral cats (81.6%; 31/38) in their yards.

No adverse events reported for any of the 100 cats in this study, regardless of treatment administered; however, two treated dogs had adverse events reported during the study. One fluralaner treated qualifying dog vomited one day before and one day after treatment administration. One sarolaner treated non-qualifying outdoor dog was found dead by its owner 7 weeks into the study (3 weeks after the second dose). The owner suspected that one of her other larger dogs killed this dog. The cause of death could not be determined as the dog’s remains were disposed of prior to reporting of this event to the study personnel. No other adverse events in treated dogs were reported.

## Discussion

A single topical dose of fluralaner provided excellent flea control in cats, achieving > 96% reduction in flea counts, a significant decrease compared to baseline, within 7 days and 100% control at 12 weeks (Table 1). The residual activity of this medication was remarkable given the constant flea re-infestation pressure from the heavily infested indoor premises and the lack of restriction on the cats from going outdoors. Faced with similar re-infestation pressure, three consecutive monthly topical applications of selamectin were significantly less effective than fluralaner for flea elimination (Table 1).

The significantly greater fluralaner efficacy over selamectin (Table 1) was also observed in comparisons between the post-treatment flea count range and the proportion of flea free cats. Cats treated once with fluralaner had no more than 4 fleas recovered in comb counts, while one cat treated with selamectin had 50 fleas on day 21 and another cat had 8 fleas at 12 weeks after 3 monthly treatments (Table 1). Within 7 days of treatment, fleas were recovered in comb counts on 31% of cats treated with fluralaner, while almost 76% of selamectin treated cats had at least one flea on day 7 (Table 1). Following 3 monthly selamectin treatments, 38.5% of cats were free of fleas at 12 weeks, significantly fewer than the 100% of cats flea free at 12 weeks following a single fluralaner treatment (Table 1).

Flea control efficacy observed following topical administration of fluralaner to cats in the present study was very similar to results of a previously published multicentric study in the United States [12] when topically administered fluralaner provided 99.1% reduction in flea counts on cats within 4 weeks and was 99.0% effective at 12 weeks. However, the selamectin flea control efficacy in this study is lower than results reported in earlier laboratory and field studies [20–23]. In a multicentric field study published in 2000, monthly application of selamectin to cats provided 92.5, 98.3 and 99.3% efficacy at days 30, 60 and 90, post-treatment, respectively [23]. In contrast, monthly selamectin efficacy in this study was only 77.8, 91.0 and 91.3% at similar post-treatment time intervals. Reasons for this efficacy reduction are likely multifactorial and possibly include: severe re-infestation pressure of cats living in subtropical Florida; slower rate of residual speed of flea kill; and/or variable susceptibility of *C. f. felis* flea strains used or encountered in previous investigations.

Significant improvement of cat dermal lesions (SCORFAD) from baseline was observed in both fluralaner and selamectin treatment groups, however, this was achieved sooner (30 days post-treatment) in fluralaner-treated cats compared to selamectin-treated cats (60 days) although treatment groups were not significantly different (Table 5). Owner assessed pruritus severity was significantly reduced from baseline on all post-treatment collection days for both treatments, and fluralaner-treated cats had significantly less severe pruritus than selamectin treated cats on days 21 and 28–30 (Table 4). Improvement in owner-assessed pruritus correlates with reductions in flea numbers and is consistent with a reduction in flea bites. Owner pruritus assessment improved faster rate in both treatment groups than dermatologic lesion improvement (Tables 4 and 5), indicating that after effective flea treatment, the reduction in pruritus severity occurs more rapidly than repair to injured and damaged skin.

The flea control efficacies of the two oral isoxazoline formulations administered to dogs in this study were very high; were not significantly different throughout the study (Table 3); and were remarkably similar to results observed in previous in-home investigations conducted in Florida [7, 24]. A single oral dose of fluralaner or three-monthly oral doses of sarolaner provided over 99% efficacy against adult fleas within 7 days of treatment and efficacy in both groups was 100% at both the 8 and 12-week assessments (Table 3). Previously, in an almost identical study design, a single oral dose of fluralaner reduced flea populations on dogs by 99.0% within 7 days and by 100% at both the 8 and 12-week post-treatment assessments [24]. Similarly, a previous Florida field study with oral sarolaner provided 99.0% reduction in flea populations on dogs at 7 days post-treatments and then further monthly doses of oral sarolaner resulted in 99.9% efficacy at 8 weeks (there was no 12 week efficacy assessment) [7]. Therefore, oral isoxazoline treatments can deliver rapid and sustained flea control for dogs in subtropical Florida.

Dogs administered oral sarolaner had significantly more fleas (geometric mean 37.9) than fluralaner treated dogs (geometric mean 26.9) prior to treatment (Table 3). However, this difference likely had no clinical effect on the study outcome because: dogs were the secondary host in this study, the efficacy of both isoxazolines in dogs was remarkably similar; efficacy of both products corresponded closely with previous in-home investigations; and pre-treatment flea counts for both groups fell within the range (20.4–51.0) of 4 recent Florida field investigations using an identical dog flea count methodology [7, 24–26].

Intermittent-light flea traps used in this investigation provide an estimate of flea biomass, flea reproduction and emergence in homes [27]. Following a single administration of fluralaner or three monthly administrations of selamectin/sarolaner to cats and dogs, 95.0% of homes in the fluralaner group and 68.8% of homes in the sarolaner/selamectin group had 0 fleas in traps at the end of the 12-week study, with both groups significantly different from baseline but not significantly different from each other (Table 3). Additionally, all 31 qualifying cats and all 18 qualifying dogs in fluralaner treated homes were flea free at the end of the study (Tables 1 and 2), showing that a single dose of fluralaner can completely eradicated fleas in these homes. All sarolaner-treated dogs were flea-free at the end of the study (Table 2); however, significantly fewer (10/26 or 38.5%) selamectin-treated cats than fluralaner treated cats were flea-free at the end of the study, despite receiving three consecutive monthly doses (Table 1). Significantly fewer (5/16 or 31.3%) selamectin/sarolaner treatment group homes achieved complete flea eradication compared to fluralaner treatment group homes (19/20 or 95.0%) (Table 3). In summary, these results show that highly effective control of fleas on both dogs and cats is essential for achieving flea control in the home.

Previous field studies have shown that halting flea reproduction leads to a shift in the sex structure of the household emergent flea population. An initially predominately female flea population will shift toward a male dominated population [25, 27]. This shift occurs because *C. f. felis* undergoes protogyny, where the first adults to emerge from a cohort of eggs are females, followed by both males and females and finally by an almost exclusively male emergent population [28]. In this study, flea sex ratio in fluralaner treatment group home traps shifted, as expected, from predominately female fleas observed during the first two weeks of the study, to a majority of male fleas between weeks 3–6, and finally to exclusively male fleas during weeks 6–12. A single male flea was the only adult collected in a trap between days 82–86 post-treatment; therefore, fluralaner treatment must have greatly reduced or possibly completely halted egg production [25, 27]. The selamectin/sarolaner treatment group home traps also had a similar shift in flea sex ratio toward a male dominated emergent adult population by weeks 3–6. However, the proportion of female fleas rebounded during weeks 8–12 with a marked increase in the proportion of female fleas collected in traps and an increase in the number of trapped fleas. This observation proves that flea reproduction was occurring in some selamectin/sarolaner homes after study week 8.

A challenge for veterinarians and pet owners is continued flea re-infestation pressure on cats and dogs from flea-infested free roaming dogs and cats and certain urban wildlife. Urban wildlife in North America, including opossums and raccoons, can be infested with *C. f. felis*. These animals move readily throughout urban environments and contaminate protected outdoor premises such as crawlspaces, decks, and under bushes with flea eggs [1, 2]. Although opossums and raccoons are nocturnal, 42.1% home owners reported observing opossums and 57.9% reported observing raccoons in their yards. In addition, almost 82% of home owners had observed cats, other than their own, in their yard. The frequent presence of potentially flea-infested hosts in areas inhabited by household dogs and cats shows that flea re-infestation pressure in this subtropical climate is substantial and continuous. Cats and dogs in this environment need to be maintained on life-long, year-round flea control to avoid the misery of flea infestation.

## Conclusions

A single topical fluralaner application to cats living in an intense flea challenge environment was more effective than three consecutive monthly doses of selamectin for flea control on cats and for flea eradication in households. For dogs, both one dose of fluralaner and three doses of sarolaner delivered a very high level of flea efficacy with no significant flea control difference between the treatments. Successful household flea control requires effective treatment of both cats and dogs in the home.

## Abbreviations

AAFP: American Association of Feline Practitioners
AAHA: American Animal Hospital Association
CAD: canine atopic dermatitis
PVAS: pruritus visual analogue score
SCORFAD: Scoring Feline Allergic Dermatitis
